# Guidelines on the management of abnormal liver blood tests

**DOI:** 10.1136/gutjnl-2017-314924

**Published:** 2017-11-09

**Authors:** Philip N Newsome, Rob Cramb, Suzanne M Davison, John F Dillon, Mark Foulerton, Edmund M Godfrey, Richard Hall, Ulrike Harrower, Mark Hudson, Andrew Langford, Anne Mackie, Robert Mitchell-Thain, Karen Sennett, Nicholas C Sheron, Julia Verne, Martine Walmsley, Andrew Yeoman

**Affiliations:** 1 National Institute for Health Research (NIHR), Birmingham Biomedical Research Centre and Centre for Liver Research, University of Birmingham, Birmingham, UK; 2 Centre for Liver Research, Institute of Immunology and Immunotherapy, University of Birmingham, Birmingham, UK; 3 Leeds Children’s Hospital, Leeds Teaching Hospitals NHS Trust, Leeds, UK; 4 Division of Molecular and Clinical Medicine, School of Medicine, University of Dundee, Dundee, UK; 5 The Pool Medical Centre, Studley, UK; 6 Department of Radiology, Cambridge University Hospitals NHS Foundation Trust, Cambridge, UK; 7 Liver4Life, Dorset, UK; 8 Public Health England, South West, UK; 9 Regional Liver and Transplant Unit, Freeman Hospital, Newcastle Upon Tyne, UK; 10 Institute of Cellular Medicine, Newcastle University, Newcastle Upon Tyne, UK; 11 British Liver Trust, Bournemouth, UK; 12 PBC Foundation, Edinburgh, UK; 13 Killick Street Health Centre, London, UK; 14 NHS Islington Clinical Commissioning Group, London, UK; 15 Clinical and Experimental Science, Faculty of Medicine, University of Southampton, Southampton, UK; 16 Chair of Trustees PSC Support, Didcot, UK; 17 Gwent Liver Unit, Royal Gwent Hospital, Newport, UK

**Keywords:** fibrosis, liver, nonalcoholic steatohepatitis, alcoholic liver disease

## Abstract

These updated guidelines on the management of abnormal liver blood tests have been commissioned by the Clinical Services and Standards Committee (CSSC) of the British Society of Gastroenterology (BSG) under the auspices of the liver section of the BSG. The original guidelines, which this document supersedes, were written in 2000 and have undergone extensive revision by members of the Guidelines Development Group (GDG). The GDG comprises representatives from patient/carer groups (British Liver Trust, Liver4life, PBC Foundation and PSC Support), elected members of the BSG liver section (including representatives from Scotland and Wales), British Association for the Study of the Liver (BASL), Specialist Advisory Committee in Clinical Biochemistry/Royal College of Pathology and Association for Clinical Biochemistry, British Society of Paediatric Gastroenterology, Hepatology and Nutrition (BSPGHAN), Public Health England (implementation and screening), Royal College of General Practice, British Society of Gastrointestinal and Abdominal Radiologists (BSGAR) and Society of Acute Medicine. The quality of evidence and grading of recommendations was appraised using the AGREE II tool. These guidelines deal specifically with the management of abnormal liver blood tests in children and adults in both primary and secondary care under the following subheadings: (1) What constitutes an abnormal liver blood test? (2) What constitutes a standard liver blood test panel? (3) When should liver blood tests be checked? (4) Does the extent and duration of abnormal liver blood tests determine subsequent investigation? (5) Response to abnormal liver blood tests. They are not designed to deal with the management of the underlying liver disease.

## Recommendations list

**Figure 1:**
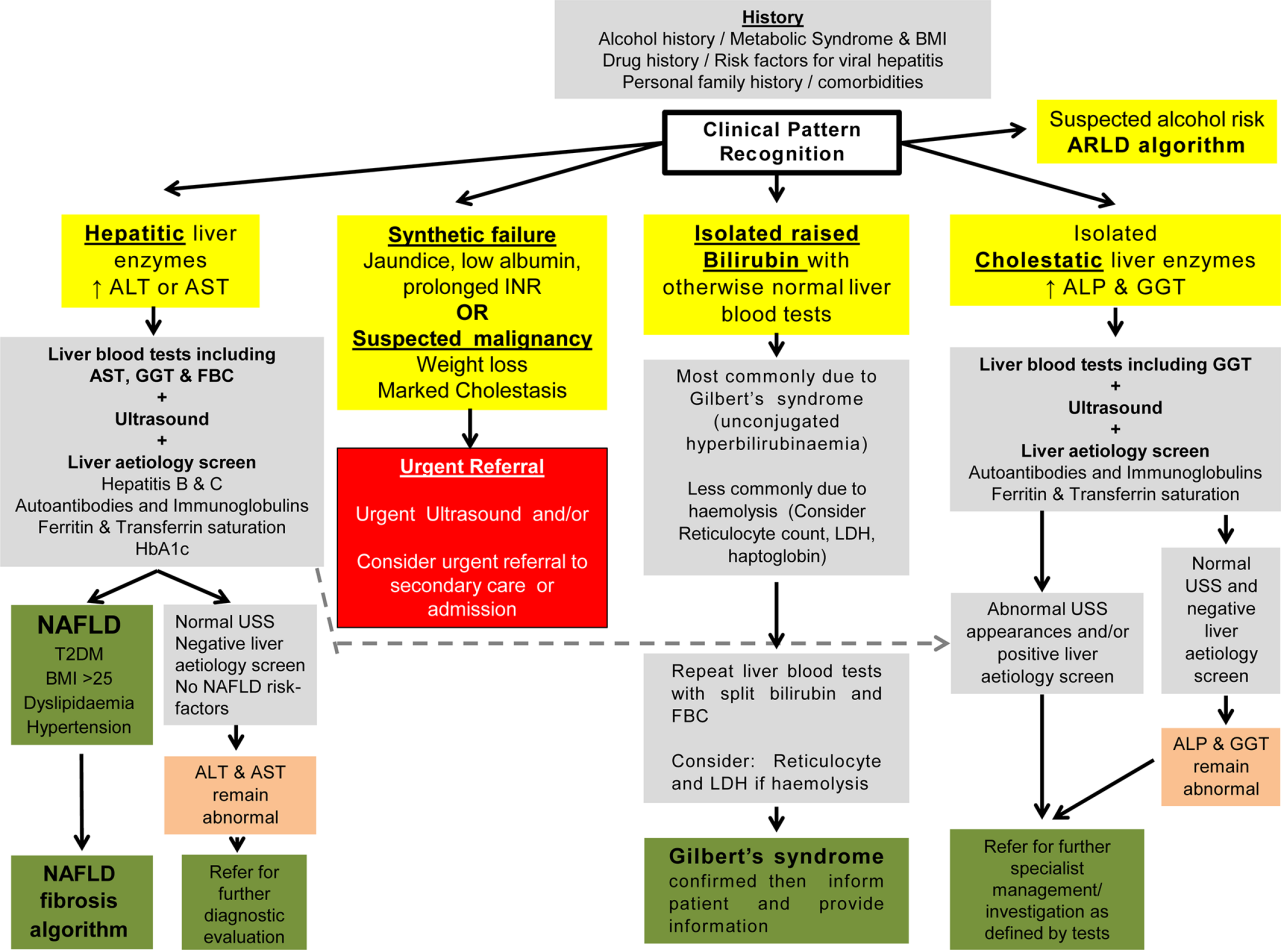
Response to abnormal liver blood tests. This figure details the initial response to abnormal liver blood tests. Boxes in yellow indicate the initial evaluation of the clinical presentation. Patients with marked derangement of liver blood tests, synthetic failure and/or suspicious clinical symptoms/signs should be considered for urgent referral to secondary care (red box). For the remainder, a clinical history alongside evaluation of the pattern of liver blood test derangement will determine choice of pathway and is shown in the grey boxes. A grey box indicates all the tests that should be requested at that stage rather than a hierarchy within it. The presence of metabolic syndrome criteria should be sought to support a diagnosis of NAFLD. For children, the text should be consulted for modification of recommendation. Areas of diagnostic uncertainty are indicated in orange boxes and the decision for repeat testing or referral to secondary care will be influenced by the magnitude of enzyme elevation and clinical context. Green boxes indicate final/definitive outcomes for users of the pathway. *Abnormal USS may well include extrahepatic biliary obstruction due to malignancy, which should result in urgent referral. ALP, alkaline phosphatase; ALT, alanine aminotransferase; ARLD, alcohol-related liver disease; AST, aspartate aminotransferase; BMI, body mass index; FBC, full blood count; GGT, γ-glutamyltransferase; INR, international normalised ratio; LDH, lactate dehydrogenase; NAFLD, non-alcoholic fatty liver disease; T2DM, type 2 diabetes mellitus; USS, ultrasound scan.

**Figure 2:**
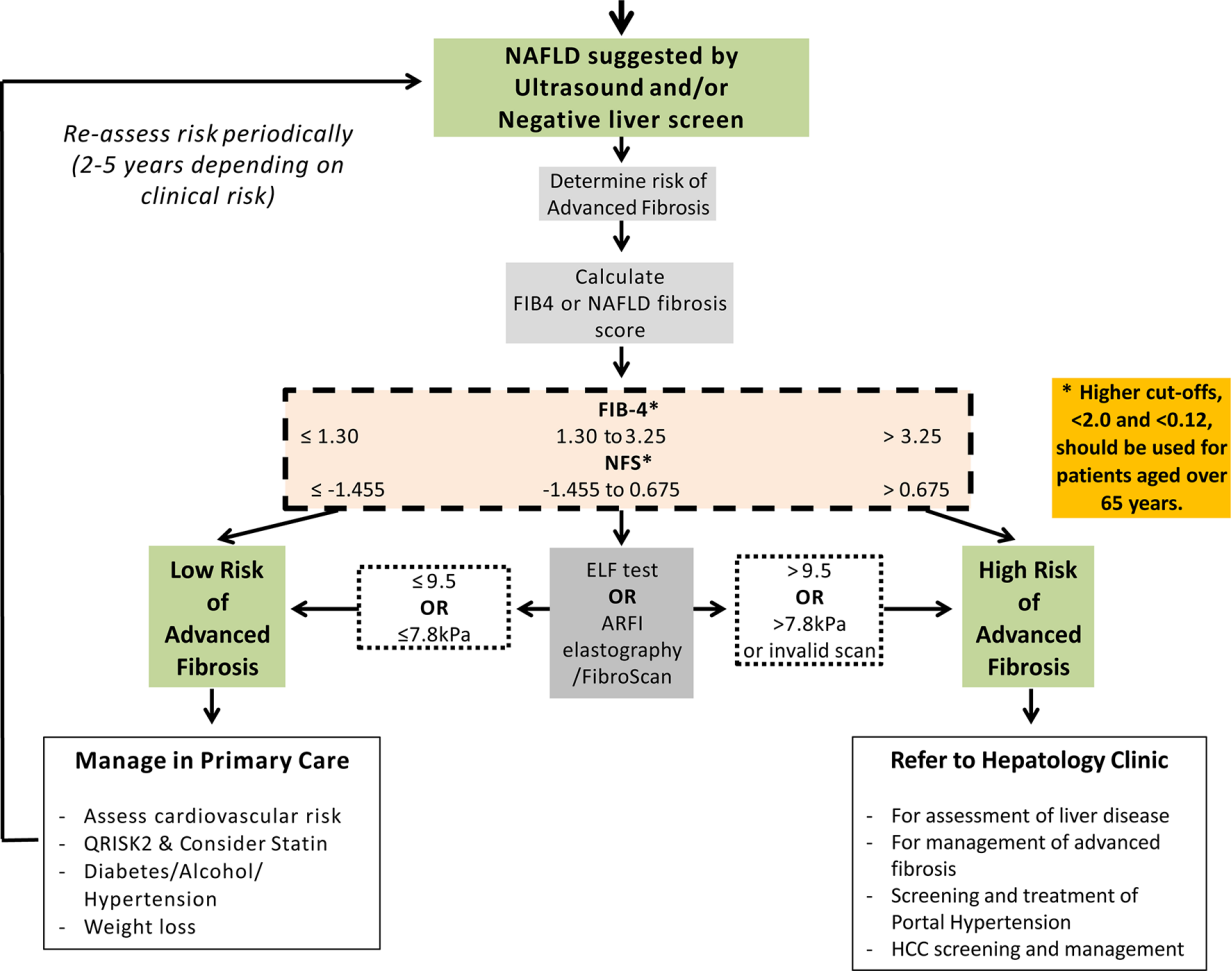
Non-alcoholic fatty liver fibrosis algorithm. For those patients with NAFLD or liver disease of unknown aetiology, the next step is to determine the likelihood of liver fibrosis. Initial assessment includes calculation of a FIB4 or NAFLD fibrosis score with values <1.3 and ≤1.455, respectively, signifying a low risk of advanced fibrosis. Higher cut-off points, <2.0 and <0.12, should be used for patients aged over 65 years. Second-line tests that should be considered include serum markers such as ELF and imaging modalities such as ARFI elastography/FibroScan. For children, the text should be consulted for modification of recommendation. Cut-off points for ARFI vary according to manufacturer and thus should be tailored to the device used. ARFI, acoustic radiation force impulse; ELF, enhanced liver fibrosis; FIB-4, fibrosis-4; HCC, hepatocellular carcinoma; NAFLD, non-alcoholic fatty liver disease; NFS, NAFLD Fibrosis Score.

- Recommendation 1: Initial investigation for potential liver disease should include bilirubin, albumin, alanine aminotransferase (ALT), alkaline phosphatase (ALP) and γ-glutamyltransferase (GGT), together with a full blood count if not already performed within the previous 12 months. (level 2b, grade B)
- Research Recommendation 1: Further evidence is required to establish the cost-effectiveness of case finding for non-alcoholic fatty liver disease (NAFLD) in high-risk groups before it can be recommended. (level 5, grade D)
- Recommendation 2: Abnormal liver blood test results should only be interpreted after review of the previous results, past medical history and current medical condition. (level 5, grade D)
- Recommendation 3: The extent of liver blood test abnormality is not necessarily a guide to clinical significance. This is determined by the specific analyte which is abnormal (outside the reference range) and the clinical context. (level 5, grade D)
- Recommendation 4: Patients with abnormal liver blood tests should be considered for investigation with a liver aetiology screen irrespective of level and duration of abnormality. Abnormal refers to an analyte which is outside the laboratory reference range (level 2b, grade B)
- Recommendation 5: In adults a standard liver aetiology screen should include abdominal ultrasound scan (USS), hepatitis B surface antigen, hepatitis C antibody (with follow-on polymerase chain reaction (PCR) if positive), anti-mitochondrial antibody, anti-smooth muscle antibody, antinuclear antibody, serum immunoglobulins, simultaneous serum ferritin and transferrin saturation. (level 2b, grade C)
- Recommendation 6: In children, ferritin and transferrin saturation may not be indicated, but autoantibody panel should include anti-liver kidney microsomal antibody and coeliac antibodies. Alpha-1-antitrypsin level and caeruloplasmin (age >3 years) should be included, and abnormalities discussed with an appropriate inherited metabolic disease specialist. (level 2b, grade C)
- Recommendation 7: Adults with NAFLD should undergo risk stratification to determine the extent of their liver fibrosis (figures 1 and 2). 
  - First-line testing should use either fibrosis-4 (FIB-4) or NAFLD Fibrosis Score (NFS) – see table 3 (level 2b, grade B). Calculation facilities for FIB-4 and NFS should be incorporated in all primary care computer systems. (level 5, grade D)
  - Second-line testing requires a quantitative assessment of fibrosis with tests such as serum enhanced liver fibrosis (ELF) measurements or Fibroscan/acoustic radiation force impulse (ARFI) elastography. (level 2b, grade B)
  - We recommend that hepatologists at a local level champion this idea and discuss it with commissioners of health to deal with the burden of liver disease in their area.
- Recommendation 8: Consider referral to alcohol services for all adults with alcohol-related liver disease (ARLD) with evidence of alcohol dependency as defined by an AUDIT score of >19. (level 3b, grade C)
- Recommendation 9: Harmful drinkers should undergo risk stratification with clinical assessment and Fibroscan/ARFI elastography. Adults should be referred to secondary care if there is evidence of advanced liver disease (features of cirrhosis or portal hypertension on imaging or from blood tests) and/or Fibroscan reading is >16 kPa (if available). (level 2b, grade B)
- Research Recommendation 2: Further evidence is required to establish the most cost-effective approach to identify patients with ARLD and NAFLD at risk of having advanced liver fibrosis.
- Recommendation 10: Adults with abnormal liver blood tests, even with a negative extended liver aetiology screen and no risk factors for NAFLD, should be referred/discussed to a gastroenterologist with an interest in liver disease/hepatologist for further evaluation (figure 1). (level 4, grade C)

## Introduction

While the number of deaths from other common conditions is falling in the UK, those due to liver disease have been increasing dramatically, with a 400% increase in the standardised mortality rate over the period 1970–2010.^1^ Notably, for those patients younger than 65, the rise in standardised mortality rate for liver disease is >500%, such that it now constitutes the fifth biggest cause of premature mortality^2^ with 64 000 years of working life lost every year.^3^ For morbidity, in England and Wales, 57 682 hospital admissions and 10 948 deaths were due to liver disease in 20 12.^1^ This rising burden of liver disease is mainly a reflection of the three the most common causes: alcohol-related liver disease, non-alcoholic fatty liver disease and viral hepatitis, although autoimmune liver disease is also a significant contributor.^4^ The burden of liver disease in children differs from that in adults, as although non-alcoholic fatty liver disease (NAFLD) is seen in all ages, reflecting the rise in childhood obesity, disease associated with injecting drug use and alcohol are rarely encountered.^5^ However, viral hepatitis is seen as a consequence of perinatal transmission, and its chronicity contributes to the disease burden seen in adults. Other causes of liver disease, such as biliary atresia or metabolic disorders,^6^ present almost exclusively in infancy or childhood, but progressive liver disease continues to evolve throughout childhood and into adulthood. There are concerted efforts to deal with this rising tide of liver disease such as the Lancet Commission on Liver Disease,^7^ the Alcohol Health Alliance and the Obesity Health Alliance.

Liver disease develops silently; there may be no signs or symptoms until the complications of liver failure or portal hypertension develop. At this late, often pre-terminal stage, the tests of liver function—bilirubin, albumin, international normalised ratio (INR) and platelet count—may be abnormal. In necro-inflammatory hepatitic diseases liver enzymes are frequently elevated,^8 9^ whereas in apoptotic diseases including fatty liver disease (alcohol and non-alcohol related), liver enzymes may be normal or elevated, but the degree of abnormality is not related to the stage of progression from simple fatty liver, through progressive fibrosis to cirrhosis.^1^ Since the current liver blood tests were developed in the 1950s, they have been the mainstay of liver disease identification, with the result that many patients with liver disease are not identified until they have developed significant liver fibrosis.^1^

Liver blood or function tests (LFTs), which are perceived to be inexpensive, are checked ever more frequently in both primary^10^ and secondary care in an attempt to exclude liver disease, for the monitoring of potential adverse effects of drugs on the liver such as statins, and for the investigation of the generally unwell patient. These tests often produce an abnormal result, the clinical significance of which is unclear. In many cases though they are requested in response to non-specific symptoms where there is little potential link between symptoms and likelihood of liver disease, or the blood tests are performed for unrelated reasons such as chronic disease monitoring.^11^ This commonly presages a cycle of additional liver blood test testing in an otherwise asymptomatic individual, and notably, most patients referred to hospital with abnormal liver tests do not have any evidence of significant liver disease.^12^ For example, University Hospital Birmingham Foundation Trust received 130 849 requests for liver blood tests in 2016, from 82 general practices and of these, 38 636 (30%) contained at least one abnormal result, defined as being outside the stated reference range. The Abnormal Liver Function Investigations Evaluation (ALFIE) study from Tayside in Scotland showed that over a 10-year period 25% of the community population aged over 16 had liver blood tests, with about a third having at least one abnormal value. Although an abnormal aspartate aminotransferase (AST) or alanine aminotransferase (ALT) level was predictive of liver disease (HR=4.2), the rate of detection was remarkably low, with only 3.9% of those with an abnormal value being diagnosed with significant liver disease within 5 years of the test.^12^ Thus, used in isolation, liver blood tests are neither specific diagnostic tools nor specific exclusion tools,^13^ whereas they can be more effectively used to assess the extent of liver fibrosis if incorporated into algorithms^14^ or used in conjunction with other modalities.^15–18^

## Guideline development

These guidelines were drafted after discussions within the liver section of the British Society of Gastroenterology (BSG) and acceptance of the proposal by the Clinical Services and Standards Committee (CSSC). There followed division of sections to be researched by designated authors and a literature review. The NICE guidelines were closely followed and guideline quality was assessed using the AGREE tool^19^ (section ‘Assessing the quality of guidelines: the AGREE II instrument’). A preliminary guideline document was drafted by the authors following discussion and, where necessary, voting by members of the Guidelines Development Group. The draft guidelines were submitted for review by the CSSC, then BSG council members. Finally, full peer review was undertaken by reviewers selected by the editor of *Gut*.

Assessing the quality of guidelines: the AGREE II instrument is an accepted method for appraising clinical guidelines.^19^ Six domains are listed:

### Scope and purpose

These guidelines are intended to be of use for all healthcare professionals, although with a major focus on the asymptomatic patient with abnormal liver blood tests. Nonetheless, the guideline will review the role/utility of liver blood tests in both symptomatic and asymptomatic patients and explore their possible role in case finding in high-risk groups or following a clinical concern. They include recommendations for both adults and children, although the evidence for children is often lacking.

No meta-analyses or randomised controlled trials concerning the management of abnormal LFTs in asymptomatic people have been carried out and therefore no grade A evidence exists in these guidelines to support the recommendations made. These guidelines are not intended to serve as rigid protocols or to replace clinical judgement.

### Guideline development group membership and stakeholder involvement

Membership of the group includes patient/patient group representation, adult and paediatric hepatologists, clinical biochemists, general practitioners, internal medicine specialists, public health specialists and radiologists.

### Rigour of development

The published literature was searched using PubMed, Medline, Web of Knowledge and the Cochrane database between October 2014 and February 2016. The Guidelines Development Group met through a series of meetings and teleconferences during that time. The level of supporting evidence (graded levels 1 to 5) is assessed by the Oxford Centre For Evidence Based Medicine (Table 1).^20^ The recommendation grade is determined on the level of evidence as follows:

**Table 1:** Evidence grading^20^

Level	Therapy/prevention, aetiology/harm	Prognosis	Diagnosis	Differential diagnosis/symptom prevalence study	Economic and decision analyses
1a	SR (with homogeneity*) of RCTs	SR (with homogeneity*) of inception cohort studies; CDR^#^ validated in different populations	SR (with homogeneity*) of level 1 diagnostic studies; CDR^#^ with 1b studies from different clinical centres	SR (with homogeneity*) of prospective cohort studies	SR (with homogeneity*) of level 1 economic studies
1b	Individual RCT (with narrow CI)	Individual inception cohort study with >80% follow-up; CDR† validated in a single population	Validating‡ cohort study with good§ reference standards; or CDR† tested within one clinical centre	Prospective cohort study with good follow-up¶	Analysis based on clinically sensible costs or alternatives; systematic review(s) of the evidence; and including multi-way sensitivity analyses
1c	All or none**	All case series or none	Absolute SpPins and SnNouts††	All or none case series	Absolute better-value or worse-value analyses
2a	SR (with homogeneity*) of cohort studies	SR (with homogeneity*) of either retrospective cohort studies or untreated control groups in RCTs	SR (with homogeneity*) of level >2 diagnostic studies	SR (with homogeneity*) of 2b and better studies	SR (with homogeneity*) of level >2 economic studies
2b	Individual cohort study (including low-quality RCT; for example, <80% follow-up)	Retrospective cohort study or follow-up of untreated control patients in an RCT; derivation of CDR†or validated on split sample‡‡ only	Exploratory‡ cohort study with good§ reference standards; CDR† after derivation, or validated only on split-sample‡‡ or databases	Retrospective cohort study, or poor follow-up	Analysis based on clinically sensible costs or alternatives; limited review(s) of the evidence, or single studies; and including multi-way sensitivity analyses
2c	‘Outcomes’ research; ecological studies	‘Outcomes’ research		Ecological studies	Audit or outcomes research
3a	SR (with homogeneity*) of case–control studies		SR (with homogeneity*) of 3b and better studies	SR (with homogeneity*) of 3b and better studies	SR (with homogeneity*) of 3b and better studies
3b	Individual case–control study		Non-consecutive study; or without consistently applied reference standards	Non-consecutive cohort study, or very limited population	Analysis based on limited alternatives or costs, poor-quality estimates of data, but including sensitivity analyses incorporating clinically sensible variations
4	Case series (and poor-quality cohort and case–control studies§§)	Case series (and poor-quality prognostic cohort studies¶¶)	Case–control study, poor or non-independent reference standard	Case series or superseded reference standards	Analysis with no sensitivity analysis
5	Expert opinion without explicit critical appraisal, or based on physiology, bench research or ‘first principles’	Expert opinion without explicit critical appraisal, or based on physiology, bench research or ‘first principles’	Expert opinion without explicit critical appraisal, or based on physiology, bench research or ‘first principles’	Expert opinion without explicit critical appraisal, or based on physiology, bench research or ‘first principles’	Expert opinion without explicit critical appraisal, or based on economic theory or ‘first principles’

*Homogeneity means a systematic review (SR) that is free from worrisome variations (heterogeneity) in the directions and degrees of results between individual studies. Not all SRs with statistically significant heterogeneity need be worrisome, and not all worrisome heterogeneity need be statistically significant.

†CDR, clinical decision rule (algorithms or scoring systems which lead to a prognostic estimation or a diagnostic category).

‡Validating studies test the quality of a specific diagnostic test based on prior evidence. An exploratory study collects information and trawls the data (eg, using a regression analysis) to find which factors are ‘significant’.

§Good reference standards are independent of the test, and applied blindly or objectively to all patients. Poor reference standards are haphazardly applied, but still independent of the test. Use of a non-independent reference standard (where the ‘test’ is included in the ‘reference’, or where the ‘testing’ affects the ‘reference’) implies a level 4 study.

¶Good follow-up in a differential diagnosis study is >80%, with adequate time for alternative diagnoses to emerge (eg, 1–6 months acute, 1–5 years chronic).

**Met when all patients died before the treatment became available but some now survive while receiving it; or when some patients died before the treatment became available but none now die while receiving it.

††An ‘absolute SpPin’: a diagnostic finding whose Specificity is so high that a Positive result rules in the diagnosis. An ‘absolute SnNout’: a diagnostic finding whose Sensitivity is so high that a Negative result rules out the diagnosis.

‡‡Split-sample validation is achieved by collecting all the information in a single tranche, then artificially dividing this into ‘derivation’ and ‘validation’ samples.

§§Poor-quality cohort study: one that failed to clearly define comparison groups and/or failed to measure exposures and outcomes in the same (preferably blinded) objective way in both exposed and non-exposed individuals and/or failed to identify or appropriately control known confounders and/or failed to carry out a sufficiently long and complete follow-up of patients. Poor-quality case–control study: one that failed to clearly define comparison groups and/or failed to measure exposures and outcomes in the same (preferably blinded) objective way in both cases and controls and/or failed to identify or appropriately control known confounders.

¶¶Poor-quality prognostic cohort study: one in which sampling was biased in favour of patients who already had the target outcome, or the measurement of outcomes was accomplished in <80% of study patients, or outcomes were determined in an unblinded non-objective way, or there was no correction for confounding factors.

- consistent level 1 studies;
- consistent level 2 or 3 studies or extrapolations from level 1 studies;
- level 4 studies or extrapolations from level 2 or 3 studies;
- level 5 evidence or troublingly inconsistent or inconclusive studies of any level.

Areas of disagreement about the recommendation grade were subjected to discussion and, if necessary, voting by members of the guidelines group. Where possible, the health benefits, side effects and risks of recommendations were discussed. The guidelines were subject to peer review after submission for consideration for publication in *Gut*.

### Clarity and presentation

Recommendations are intended to be specific to particular situations and patient groups; where necessary, different options are listed. Where the evidence and recommendation is restricted to adults, this will be stated. The term ‘patients’ implies all ages. Key recommendations are linked to discussion threads on a discussion forum hosted on the BSG website.

### Applicability

We have discussed organisational changes that may be needed in order to implement these recommendations with the British Liver Trust, the British Association for the Study of the Liver, the British Society of Gastroenterology, the Royal College of General Practice, the Specialist Advisory Committee in Clinical Biochemistry/Royal College of Pathology and Association for Clinical Biochemistry, the British Society of Paediatric Gastroenterology, Hepatology and Nutrition (BSPGHAN), Public Health England, the British Society of Gastrointestinal and Abdominal Radiologists (BSGAR) and the Society of Acute Medicine. We have attempted to identify key criteria for monitoring and audit purposes.

### Editorial independence and conflict of interest

Guideline group members have declared any conflicts of interest. There is full editorial independence from the BSG, which commissioned the guideline. The guideline was subsequently peer reviewed by the CSSC, who provided comments and suggestions.

## Scheduled review of guidelines

The proposed time for review of the guidelines is 5 years to take into account new developments. To ensure that there is a facility for feedback after publication, links to the BSG discussion forums corresponding to the particular section of these guidelines are included with this document. Feedback from general practitioners will also be incorporated—for example, via the newly established British Liver Trust/Royal College of General Practitioners (RCGP) clinical priority programme. In accordance with the AGREE II tool the BSG forum will provide feedback.

## What constitutes a liver blood test?

Liver blood tests are readily available biochemical laboratory tests, with the standard panel varying from hospital to hospital.^21^ They have historically been referred to as LFTs, yet the predominant abnormality relates not to liver dysfunction, but to elevations of hepatobiliary liver enzymes. For this reason this guideline will refer to liver blood tests and not LFTs as it more accurately captures their usage in clinical practice. Hepatobiliary enzymes, when interpreted in isolation convey information on the level of ongoing injury, whereas bilirubin, albumin and INR convey information on liver function, with platelets conveying information on the level of fibrosis. In this guideline an abnormal liver blood test is defined as being a value outside the standard reference interval, although there is an emerging literature suggesting that the current reference intervals for ALT may be too high.^22 23^

**Bilirubin** is predominantly the by-product of the breakdown of the haem component of haemoglobin by the reticuloendothelial system.^24^ It exists in two forms, unconjugated and conjugated. Bilirubin is transported to the liver in its insoluble unconjugated form, where it is converted into soluble conjugated bilirubin in order to be excreted. Unconjugated hyperbilirubinaemia is usually due to haemolysis or impaired conjugation whereas conjugated hyperbilirubinaemia is typically due to parenchymal liver disease or obstruction of the biliary system.

Most laboratories will routinely report total bilirubin, which comprises unconjugated and conjugated fractions. Elevations of either fraction will therefore lead to a rise in the measured bilirubin concentration. The most common cause of an isolated elevated bilirubin concentration is Gilbert’s syndrome, which is an inherited disorder of metabolism and leads to impaired conjugation via reduced activity of the enzyme glucuronyltransferase.^25^

Except in the neonatal period, the majority of measurable bilirubin should be conjugated, even in individuals with significant liver disease. Hence if the majority of the elevated bilirubin comprises the unconjugated fraction then the cause, in the absence of haemolysis, is virtually always Gilbert’s syndrome. As Gilbert’s syndrome is not associated with liver disease or ill health, any such individuals should be fully reassured.^26^ In the neonatal period, there may be a physiological increase in total bilirubin, which is unconjugated. This may be pathological if high or prolonged.^27^ In neonates and infants in whom the conjugated bilirubin is >25 μmol/L, referral to a paediatrician for urgent assessment of possible liver disease is essential.^28 29^

**Albumin** is a protein that is produced only in the liver and has multiple biological actions, including maintenance of oncotic pressure, binding of other substances (such as fatty acids, bilirubin, thyroid hormone and drugs), metabolism of compounds, including lipids, and antioxidant properties. As albumin is only produced by the liver, the serum albumin concentration is often considered as a marker of the synthetic function of the liver. However, overinterpretation of the measured concentrations of albumin as a marker of the severity of liver disease is not always merited. Albumin concentrations are reduced in many clinical situations, including sepsis, systemic inflammatory disorders, nephrotic syndrome, malabsorption and gastrointestinal protein loss.

**Prothrombin time (PT) and INR** are assessments of blood clotting, which are used to measure liver function, as the underlying protein clotting factors (II, V, VII, IX and X) are made in the liver. If there is significant liver injury (usually loss of >70% of synthetic function), this results in a reduction in clotting factor production and subsequent coagulopathy, as confirmed by a prolonged PT or INR. While a prolonged PT/INR can indicate either acute or chronic liver dysfunction it can also be caused by vitamin K deficiency as seen in fat malabsorption and chronic cholestasis.

A reduction in **platelets,** termed thrombocytopenia, is the most common haematological abnormality found in patients with chronic liver disease and is an indicator of advanced disease. Multiple factors culminate in a low platelet count: decreased production, splenic sequestration and increased destruction. Decreased production is a consequence of bone marrow suppression, as caused by alcohol, iron overload, drugs and viridae, and also by a reduction in thrombopoietin levels in chronic liver injury. Splenic sequestration results from hypersplenism, which is a consequence of portal hypertension seen in advanced liver fibrosis. Platelet destruction is also increased non-specifically in liver cirrhosis owing to shear stress, fibrinolysis and bacterial translocation, whereas in specific causes of autoimmune liver disease, immunologically mediated destruction of platelets occurs owing to antiplatelet immunoglobulin.

**Alkaline phosphatase** (ALP) is produced mainly in the liver (from the biliary epithelium) but is also found in abundance in bone and in smaller quantities in the intestines, kidneys and white blood cells. Levels are physiologically higher in childhood, associated with bone growth, and in pregnancy due to placental production. Pathologically increased levels occur mainly in bone disease (eg, metastatic bone disease and bone fractures) and cholestatic liver disease—for example, primary biliary cholangitis, primary sclerosing cholangitis, common bile duct obstruction, intrahepatic duct obstruction (metastases) and drug-induced cholestasis. Furthermore, hepatic congestion secondary to right-sided heart failure can also lead to cholestasis (elevated ALP levels and/or bilirubin).

When ALP is elevated in isolation, the measurement of γ-glutamyltransferase can indicate whether the ALP is of hepatic or non-hepatic origin.^30^ While there are no data on the most likely causes of an isolated raised ALP in an asymptomatic population, the the most common cause is likely to be vitamin D deficiency, or normal increase seen in childhood due to rapid growth. Other causes include Paget’s disease and bony metastases. If doubt still exists, the use of electrophoresis to separate the isoenzymes of ALP can differentiate hepatic from non-hepatic causes of increased ALP.

**AST and ALT** are enzymes present in hepatocytes and are released into the blood stream in response to hepatocyte injury or death (hepatitis). Elevations in either of these enzymes are the the most common abnormality seen on liver blood test profiles. Both enzymes are present in many differing types of tissue, but ALT is considered more liver-specific since it is present in low concentrations in non-hepatic tissue, and non-liver related elevations are uncommon. However, AST is abundantly present in skeletal, cardiac and smooth muscle and so may be elevated in patients with myocardial infarction or myositis. Although ALT is considered a more specific indicator of liver disease, the concentration of AST may be a more sensitive indicator of liver injury in conditions such as alcohol-related liver disease and in some cases of autoimmune hepatitis (AIH).^31 32^ In children, creatine kinase measurement may help to determine whether an isolated rise in ALT or AST is due to an underlying skeletal muscle disorder, such as muscular dystrophy.

**γ-Glutamyltransferase** (GGT) is abundant in the liver and also present in the kidney, intestine, prostate and pancreas but not in bone; therefore it can be useful in confirming that an elevated ALP is of liver and not bony origin.^33^ GGT is most commonly elevated as a result of obesity, excess alcohol consumption or may be induced by drugs. Although an elevated GGT has a low specificity for liver disease, it is one of the best predictors of liver mortality.^12^ It is particularly useful in children to establish the likelihood of biliary disease when ALP is not a reliable indicator. Predominant causes of cholestasis in children include congenital abnormalities of the biliary tract and genetic disorders affecting bile synthesis and excretion.

## What constitutes a standard liver blood test panel?

There are remarkably few data to determine what an optimal liver blood test panel should include, although this would be influenced by the clinical setting.^34^ The Health Technology Assessment commissioned Birmingham and Lambeth Liver Evaluation Testing Strategies (BALLETS) study reported that ALT and ALP identified the vast majority of adults with necro-inflammatory liver disease. The routine addition of GGT led to a marginal increase in sensitivity but at the cost of a loss of specificity and a higher false-positive rate.^11^ But the analysis did not include adults with NAFLD or alcohol-related liver disease (ARLD), which account for 90% of liver mortality,^1^ in whom liver blood tests and the follow-on liver aetiology screen are seldom diagnostic. In this setting GGT and AST would aid the sensitivity of detecting such patients. Addition of GGT to a liver blood test panel increases the likelihood of an adult having abnormal liver blood tests from around 15% to 30%^35^ and, notably, a raised GGT is associated with increased liver as well as all-cause (including cancer) mortality, with the greatest risk being observed in those with the most significant elevations of GGT.^12 36 37^ In addition, the routine addition of AST to the initial panel did not improve the detection of specific disease.^11^

The analysis from the BALLETS study was predicated on the identification of adults with established causes of liver disease such as autoimmune liver disease, viral hepatitis or metal storage disorders, which were found in just 5% of those with abnormal liver blood tests.^11^ Thus, these data would support a strategy of a streamlined panel with high sensitivity without generating large numbers of false positives, which have the potential to lead to greater patient anxiety, overinvestigation and considerably increased costs.

***Recommendation 1:* *Initial investigation for potential liver disease should include bilirubin, albumin, ALT, ALP and GGT, together with a full blood count if not already performed within the previous 12 months. (level 2b, grade B)***

If there is clear indication of a specific clinical risk—for example, in high-risk groups such as injecting drug users, migrants from high prevalence areas or prisoners, then some aspects of second-line testing can be undertaken simultaneously. In many patients with liver damage an assessment of liver fibrosis is critical in making decisions about referral and management. In adults, clues to the level of liver fibrosis can be gleaned from the use of non-invasive algorithms such as the AST to ALT ratio.^12^ An AST:ALT ratio of >1 indicates advanced fibrosis/cirrhosis,^38^ hence the inclusion of this ratio in algorithms has the potential to assess the risk of significant fibrosis in adults with abnormal liver blood tests. However, non-invasive markers have not been sufficiently validated in children to be routinely applied in clinical practice.

An important consideration when evaluating the risk of hepatic fibrosis is that both AST and ALT can be normal even in the setting of cirrhosis, and the utility of the AST:ALT ratio in adults persists even if both values are within the normal reference interval.^39^ While it is hard to justify the routine analysis of both AST and ALT together on every liver blood test request, a strategy not supported by the data from the BALLETS study, subsequent testing of AST (or ALT depending which one is undertaken first) to calculate the AST:ALT ratio is clearly desirable. From a patient and cost perspective this is likely to be more cost-effective if performed by ‘reflex’ on the same sera following the detection of an abnormal ALT or GGT. To date there is no firm evidence that this is a cost-effective approach, although the results of a pilot study of such ‘reflex’ testing and additional up-front aetiology screen testing from Wales and Scotland are awaited.

## When should liver blood tests be checked?

There are a range of settings where requesting liver blood tests should be considered to determine the presence, or severity, of liver disease: 

### Non-specific symptoms

Liver disease tends to develop silently with no signs or symptoms, and there is evidence that the majority of people with late-stage liver disease are undiagnosed.^6^ However, inflammatory liver diseases including autoimmune liver disease and viral hepatitis can be associated with symptoms. For example, 75% of patients with AIH have one or more non-specific symptoms, such as fatigue, nausea or anorexia.^7^ These diseases can be effectively treated, and are often diagnosed late, so the presence of these non-specific symptoms would be an indication to check routine liver blood tests, accepting that there are many other causes for these symptoms.

### Evidence of chronic liver disease

Patients with symptoms or signs of cirrhosis, portal hypertension or liver failure, including ascites, peripheral oedema, spider naevi and hepatosplenomegaly, need liver blood tests to monitor their function. In that regard the inclusion of INR is important to fully define their synthetic function.

### Conditions which are associated with a high risk of developing liver disease

Autoimmune liver disease is more common in patients with pre-existing autoimmune diseases, and liver blood tests may be appropriate if clinical symptoms change to suggest development of liver disease—for example, pruritus in primary biliary cholangitis. Patients with inflammatory bowel disease (including ulcerative colitis and Crohn’s disease) have a particular notable risk of developing the autoimmune cholestatic liver disease, primary sclerosing cholangitis; disease prevalence is estimated at just under 10%.^40^ Primary sclerosing cholangitis-inflammatory bowel disease is associated with increased complications relating to liver disease, as well as increased colorectal cancer risk.^41^ Periodic monitoring of liver blood tests is therefore common practice, with a low clinical threshold for investigation of cholestatic liver blood tests by MRI. In the absence of currently approved medical therapy ongoing efforts clinically focus on early recognition of disease with subsequent risk stratification, in order to facilitate timely consideration of trial-based intervention.

### Use of hepatotoxic drugs

A wide variety of drugs are associated with liver disease, and a requirement for the monitoring of liver functions may be documented.^8^ In this systematic review the drugs most commonly implicated included: carbamazepine, methyldopa, minocycline, macrolide antibiotics, nitrofurantoin, statins, sulfonamides, terbinafine, chlorpromazine and methotrexate. In addition, drugs can cause fatty liver and steatohepatitis and vascular injury. Methotrexate treatment requires special care, to prevent dose-dependent liver fibrosis, and non-invasive markers of fibrosis should be monitored.^9–11^ Although statins can lead to drug-induced liver injury, this is very rare, with studies demonstrating they are safe in patients with pre-existing abnormal liver enzymes.^42^

On occasions it can be difficult to establish the relative contribution of a drug or drugs alongside possible concomitant liver disease. In this situation clinical judgement needs to be exercised to determine what is the major contributor and the need to discontinue medication. This will be influenced by the pattern of liver blood tests, the timing of medication use with respect to the liver blood abnormality developing and the clinical setting.

### Family history of liver diseases

Investigating the relatives of patients with familial diseases, including haemochromatosis or Wilson’s disease, would be an indication for the specific relevant tests: ferritin and transferrin saturation, haemochromatosis genotype, caeruloplasmin and urinary copper.

Liver enzymes are a poor guide to the development of progressive liver fibrosis in alcohol-related liver disease, but elevated enzymes, of which GGT is the best predictor of mortality,^12^ can be useful aids to behaviour change.^13^ The current NICE recommendation is to screen for advanced liver disease using Fibroscan in patients drinking at harmful levels (50 units/week in men and 35 units/week in women), and there is emerging evidence that a diagnosis of liver fibrosis can be an effective stimulus for behaviour change.^14^

### Viral hepatitis

Viral hepatitis may be associated with non-specific symptoms, including fatigue, which may be severe,^15^ but the majority of patients are symptom free and identified as a result of risk factors, including country of origin or parental exposure. While liver blood tests can give an indication of necro-inflammation or of advanced fibrosis, a key early test is serology for viral hepatitis in high-risk groups, such as people who inject drugs, migrants from high-prevalence areas, prisoners, as liver blood tests can be normal in this setting (Table 2).

**Table 2:** Liver aetiology table for patients with non-acute abnormal liver blood tests

	Standard liver aetiology panel	Extended liver aetiology panel
Viral hepatitis	Hepatitis B surface antigen AND hepatitis C antibody (with follow-on PCR if positive)	Anti-HBc and anti-HBs hepatitis B DNA quantification of hepatitis delta in high-prevalence areas
Iron overload	Ferritin AND transferrin saturation	Haemochromatosis gene testing
Autoimmune liver disease (excluding PSC)	Anti-mitochondrial antibody, anti-smooth muscle antibody, antinuclear antibody, serum immunoglobulins	Anti-LKM antibody and coeliac antibodies (consider ANCA in the presence of cholestatic liver blood tests)
Metabolic liver disease		Alpha-1-antitrypsin level; thyroid function tests; caeruloplasmin (age >3 and <40 years)±urinary copper collection

ANCA, antineutrophil cytoplasmic antibodies; LKM, liver kidney miscrosome; PCR, polymerase chain reaction; PSC, primary sclerosing cholangitis.

### Presence of lifestyle risk factors associated with the development of NAFLD: obesity/type 2 diabetes

Commonly, the question about non-alcoholic fatty liver arises in response to the incidental observation of abnormal liver blood tests or an echobright liver on an ultrasound scan (USS). Case finding or screening to identify patients with NAFLD remains controversial, with conflicting advice from American^43^ and European^44^ guidelines. Indeed recent NICE guidance does not advocate this at present, although the advent of new diagnostics and treatments allied with cost-effectiveness analyses may affect this in the future.

***Research Recommendation 1: Further evidence is required to establish the cost-effectiveness of case finding for NAFLD in high-risk groups before it can be recommended. (level 5, grade D)***

## Does the extent and duration of abnormal liver blood tests determine subsequent investigation?

Many previous guidelines have stipulated minimum criteria for the extent and duration of any liver blood test abnormality before further investigation is considered, which in the main has been influenced by workload considerations given the large numbers of liver blood tests outside the standard reference intervals. However, over 50% of patients presenting with end-stage liver disease, without a previous diagnosis of chronic liver disease, were previously noted to have abnormal liver blood tests in their health records, indicating inadequate investigation.^12^ The Epidemiology of Liver Disease in Tayside (ELDIT) project used electronic case record linkage to diagnose liver disease and demonstrated that 20% of all liver blood tests measured were found to be abnormal, with <10% of these explained by existing liver disease. Thus, GPs and practice nurses who request liver blood tests have the problem of managing the 20% patients who have test results reported as ‘abnormal’ and outside the laboratory reference intervals. GP management strategies can vary from ignoring some results (potentially unsafe), repeating them (inconvenient to the patient), requesting more tests (expensive) or referring patients to a gastroenterologist (incurring NHS costs). The NHS faces the potential for a significant rise in the costs and consequences of the uncertainty GPs have in managing liver blood test results, necessitating clear guidance on how to respond to these tests.

## Importance of context

Interpretation of abnormal liver blood tests requires an understanding of the context in which they arise. This can be illustrated in the extreme by a patient receiving statin therapy who has an ALT of 80 U/L, who is well and requires continued treatment with the statin compared with a patient with end-stage alcohol-related liver disease with an ALT in the normal reference interval at 30 U/L and who may have a life expectancy of weeks. A common assumption is that the detected abnormality represents the first presentation of abnormal LFTs, when it should be standard practice to review previous blood test records and past/current medical history before requesting additional investigations and referrals.

Another setting in which liver bloods are commonly abnormal but not necessarily of clinical concern is pregnancy where the alkaline phosphatase and serum albumin are often elevated and reduced, respectively. Other changes in liver bloods in this setting may indicate worsening of pre-existing disease or the development of pregnancy-related disease, which would warrant prompt investigation.^45^

***Recommendation 2: Abnormal liver blood test results should only be interpreted after review of the previous results, past medical history and current medical condition. (level 5, grade D)***

## Extent of abnormality

It is assumed that the magnitude of derangement of a liver blood test panel correlates with prognosis, and for this reason threshold values above the upper limit of the reference interval are commonly used when directing the need for further investigation. However, this assumption is not supported by the literature, and prognosis is more clearly determined by diagnosis and context within which the tests are requested. To illustrate this consider two patients; a patient with an acute hepatitis A infection can have ALT values >1000 U/L, whereas a patient with hepatitis C can have an ALT within the normal reference interval, yet 10 years later the patient with hepatitis A is likely to be alive and well irrespective of how they are managed, whereas the patient with hepatitis C if not investigated and diagnosed is at substantial risk of progressing to end-stage liver disease. Indeed, the the most common causes of abnormal liver blood tests leading to chronic liver disease—namely non-alcoholic fatty liver disease, alcohol-related liver disease and hepatitis C, are frequently associated with only mild or moderate liver blood test abnormalities. Therefore, despite the increasing use of liver blood tests, patients continue to present with undiagnosed end-stage liver disease, which might have been preventable by earlier diagnosis.

Moreover, the current upper limit of normal for many of the liver enzymes (for example ALT) may be too high, which is probably a consequence of patients with occult NAFLD being included in the generation of normal serum ALT ranges.^22^ This is perhaps best appreciated in patients with chronic hepatitis B, where treatment guidelines recommend an ALT of >30 U/L as being significant in males and >19 U/L significant for females. Further, indirect evidence for this comes from the recognition that in some patients with autoimmune hepatitis their fibrosis stage progresses despite apparent control of their inflammatory process via perceived normal aminotransferase levels. This is compounded by the knowledge that many patients with significant liver fibrosis may have liver enzymes in the normal reference range and normal synthetic function, increasing the difficulty of their early identification. Thus, the clinical assessment of such individuals is critical in determining what the question is (do they have fibrosis?), which tests should be ordered and how should they be interpreted.

***Recommendation 3: The extent of liver blood test abnormality is not necessarily a guide to clinical significance. This is determined by the specific analyte which is abnormal (outside the reference range) and the clinical context. (level 5, grade D)***

## Duration of abnormality and retesting

As with extent of liver blood test derangement, there are also assumptions that the duration is a reflection of clinical significance, thus necessitating routine repeat testing for patients with mildly abnormal liver blood tests. This is predicated on the belief that many liver blood test abnormalities may be transient and incidental and will normalise thus precluding any significant liver disease. While this may be true of some acute liver diseases, it is manifestly not the case for many chronic liver diseases such as HCV and NAFLD where even normalised liver blood tests do not necessarily imply absence or resolution of disease.

Moreover, as demonstrated by the BALLETS study, 84% of adults still had abnormal tests when repeated 1 month later.^11^ When repeating blood tests (to see if they have normalised) the whole cost of the investigation must be borne in mind, which includes recalling the patient as well as obtaining and transporting the blood sample to the laboratory and the cost of the laboratory analysis. Therefore, a strategy of simply repeating abnormal tests can only be justified where there is a high degree of certainty that the abnormality will resolve in response to an identified acute insult. In other cases, detection of the first abnormality should trigger investigation of the aetiology, or repeat testing to assess progression or disease severity where there is a suspicion that the underlying cause may require urgent referral/admission.

The Health Technology Assessment-commissioned ALFIE study, which was a retrospective study of outcomes following abnormal liver blood tests in patients over 16 years of age seen in primary care, demonstrated that just 50% of abnormal liver blood tests were ever followed up.^12^ This highlights the challenges in identifying/capturing significant liver disease early and emphasises the importance of assessing such patients expediently without adding unnecessary delays.

***Recommendation 4: Patients with abnormal liver blood tests should be considered for investigation with a liver aetiology screen irrespective of level and duration of abnormality. Abnormal refers to an analyte which is outside the laboratory reference range. (level 2b, grade B)***

## Clinical pattern recognition for liver blood tests

There are three common patterns of abnormal liver test results whose recognition can aid diagnosis:

1. **Isolated raised bilirubin**—most commonly caused by Gilbert’s syndrome (affects 5–8% of the population).^46^ Consider haemolysis in patients with anaemia. Repeat liver blood tests on a fasting sample with a full blood count and a direct and indirect bilirubin; the total bilirubin should rise further, owing to the indirect component, and there should be no evidence of anaemia. If the patient is anaemic, haemolysis needs to be excluded by requesting reticulocyte count/lactate dehydrogenase/haptoglobin. If the unconjugated bilirubin is more markedly elevated (>40 μmol/L) then rarer causes such as Crigler-Najjar syndrome^46^ should be considered and genetic testing undertaken.
2. **Cholestatic**—predominantly raised ALP and GGT indicate cholestasis. Common causes include primary biliary cholangitis, PSC, biliary obstruction (stones, strictures, neoplasia, etc), hepatic congestion and drug-induced liver injury. In children, additional disorders that may present with cholestasis include biliary tract abnormalities and genetic disorders of bile synthesis and excretion. However, an isolated raised ALP may be caused by vitamin D deficiency and not be liver related, or it may relate to raised values during periods of rapid growth in childhood, and thus the presence of a concomitantly elevated GGT can help confirm the cause of liver disease. In children with specific inherited disorders of bile acid synthesis and transport, however, GGT is characteristically low or normal. In these disorders, cholestasis occurs without GGT elevation.
3. **Hepatitic**—predominantly raised ALT and AST indicate hepatocellular liver injury (hepatitis). Common causes include viral hepatitis, NAFLD, ARLD, AIH and drug-induced liver injury. Details of the approach to these liver blood test abnormalities are given in the subsequent section on outcomes and pathways.

## Response to abnormal liver blood tests: outcomes and pathways￼

As indicated in figure 1 the presence of unexplained clinical jaundice or suspicion of possible hepatic or biliary malignancy should lead to an immediate referral. In all other adults with incidentally raised liver enzymes it is important to take a careful history and perform a targeted clinical examination to look for the cause. Liver enzymes can occasionally be raised owing to intercurrent illness, although when liver blood tests were repeated, 84% of tests remained abnormal on retesting after 1 month, and even at 2 years 75% remained abnormal.^11^ Thus, in a patient with abnormal liver blood tests it is not recommended to simply repeat the same panel of tests but to determine the cause unless there is a high index of clinical suspicion that it is a transient finding. In children, there should be a low threshold for referral to a paediatrician for further investigation, as the most common causes of liver dysfunction in adults are less common in children, and there is a wider differential diagnosis.

Therefore, the response to the finding of abnormal liver blood tests should be to obtain a thorough clinical history, including age; ethnicity/country of birth (to explore possible risk of hepatitis B or C); specific symptoms (jaundice, abdominal pain, weight loss, pruritus, etc); comorbidity; drug history (prescribed, over the counter, herbal, injecting drug use, illicit); travel history; occupational exposure; tick bites; muscle injury; alcohol history (current and past intake in average units per week, consider AUDIT C); features of the metabolic syndrome (central obesity, hypertension, diabetes/insulin resistance and dyslipidaemia); family history; other symptoms, and, additionally, in children a maternal, neonatal, nutritional and developmental history. For patients with more marked elevations in ALT (>1000 U/L) other possible causes of viral hepatitis should be considered, including hepatitis A and E and cytomegalovirus. Examinations should include: body mass index and an abdominal examination looking for hepatosplenomegaly, ascites and other signs of chronic liver disease. PSC should be considered for patients with raised cholestatic liver enzymes and a personal or family history of autoimmune disease or personal history of inflammatory bowel disease. No diagnostic or serological markers exist for PSC and ￼MRI may be required at the outset.

Investigations should include a standard liver aetiology screen or core panel (table 2) to identify the cause of damage and exclude additional pathologies. There is uncertainty as to whether the entire extended liver aetiology screen should be undertaken in response to abnormal liver blood tests, but in most situations only the core panel should be performed with the extended panel (table 2) reserved for patients with no clear cause. The choice of blood tests in the core panel is influenced by prevalence (BALLETS) in the UK and the identification of treatable causes of liver disease.

Patients with evidence of hepatitis B (HBsAg positive), HCV (antibody positive then PCR positive), autoimmune hepatitis (raised IgG ± positive autoantibodies), primary biliary cholangitis (cholestatic liver enzymes+positive anti-mitochondrial antibody), PSC (cholestatic liver enzymes ± history of inflammatory bowel disease) or haemochromatosis (raised ferritin and transferrin saturation >45%) should be referred to a specialist clinic in accordance with locally agreed guidance. An isolated elevated serum ferritin result is commonly seen in dysmetabolic iron overload syndrome as found in the setting of alcohol excess, NAFLD and other chronic liver diseases and does not reflect haemochromatosis. The presence of dilated bile ducts requires further assessment and consideration of urgent hospital referral depending on the clinical setting. In the BALLETS study,^11^ in a cohort of 1290 adults in primary care, fully characterised and followed up for 2 years, <5% of people with abnormal liver blood test results had a specific disease affecting the liver. In only 1.3% was a specific liver disease identified requiring immediate treatment (13 with viral hepatitis and four genetic haemochromatosis). Notably, the country of origin (not ethnic group) was the strongest predictor of viral hepatitis.^47^ The condition of infants with neonatal cholestasis (conjugated bilirubin >25 μmol/L) should be discussed urgently with the local paediatrician.

***Recommendation 5: In adults a standard liver aetiology screen should include abdominal USS, hepatitis B surface antigen, hepatitis C antibody (with follow-on polymerase chain reaction if positive), anti-mitochondrial antibody, anti-smooth muscle antibody, antinuclear antibody, serum immunoglobulins, simultaneous serum ferritin and transferrin saturation. (level 2b, grade C)***

***Recommendation 6: In children, ferritin and transferrin saturation may not be indicated, but autoantibody panel should include anti-liver kidney microsomal antibody and coeliac antibodies. Alpha-1-antitrypsin level and caeruloplasmin (age >3 years) should be included, and abnormalities discussed with an appropriate inherited metabolic disease specialist. (level 2b, grade C)***

Nearly 4 in 10 adults had a ‘fatty liver’ on ultrasound in the BALLETS study, and an abnormal ALT concentration was the strongest laboratory predictor of this finding.^11^ Obesity was more strongly associated with ‘fatty liver’ than with alcohol excess, but one-quarter of adults with ‘fatty liver’ were neither overweight nor excessive alcohol drinkers. The majority of adults with abnormal liver blood tests will be identified as having NAFLD or ARLD and most will not need referral to a specialist, but will require reinforcement of lifestyle advice and ongoing assessment in primary care. For such patients, together with those with other aetiologies, it is important to establish if there is significant liver fibrosis and risk of progression of cirrhosis,^48^ as early recognition of liver disease and appropriate treatment can prevent progression to end-stage liver disease. As illustrated in figures 1 and 2, this can be achieved in adults by use of algorithms and non-invasive fibrosis markers, with recourse to specialist clinics and liver biopsy as needed. A range of non-invasive algorithms has been examined in NAFLD^14^ and ARLD but in this guideline we will focus on those with the greatest evidence base.

## Approach to common conditions

### NAFLD

NAFLD is diagnosed by the presence of an echobright liver on ultrasound in the absence of excessive alcohol consumption. For adults with NAFLD it is recommended that a first-line, non-invasive assessment, such as Fibrosis-4 (FIB-4)^49^ or NAFLD Fibrosis Score (NFS),^50^ is undertaken to identify patients with advanced fibrosis (table 3). Patients with a low FIB-4 (<1.3 for those aged <65 years or <2.0 for those >65 years) or low NFS (<−1.455 for those aged <65 years or <0.12 for those >65 years) can be managed in primary care.^51^ Presently, the mainstay of treatment for NAFLD is to reduce calorie intake and increase physical activity with the aim of inducing gradual and long-term weight loss (see figures 1 and 2).

**Table 3:** Non-invasive algorithms for gauging liver fibrosis in patients with NAFLD

NAFLD fibrosis score	−1.675 + 0.037 × age (years) + 0.094 × BMI (kg/m^2^) + 1.13 × IFG/diabetes (yes=1, no=0) + 0.99 × AST/ALT ratio – 0.013 × platelet (×10^9^/L) – 0.66 × albumin (g/dL) www.nafldscore.com
Fibrosis-4 (Fib-4)	(age × AST)/(platelets × (√ALT)) https://gps.camdenccg.nhs.uk/fib-4-calculator

ALT, alanine aminotransferase; AST, aspartate aminotransferase; BMI, body mass index; IFG, impaired fasting glucose; NAFLD, non-alcoholic fatty liver disease.

Those patients with indeterminate FIB-4 (1.3–3.25) or NFS scores (−1.455 to 0.675) should undergo further testing with a second-line test such as serum enhanced liver fibrosis (ELF)^15 16 52^ or Fibroscan/acoustic radiation force impulse (ARFI) elastography.^18 53–56^ The decision to use Fibroscan or ARFI should be based on local expertise and availability of respective devices. Consider referral to a specialist clinic for patients with serum ELF measurements >9.5 or Fibroscan >7.8 kPa. Cut-off points for ARFI vary according to the manufacturer^57–61^ and thus should be tailored to the device used.^56^

Finally, those patients with elevated FIB-4 (>3.25) or NFS (>0.675) should be considered for referral to a specialist clinic irrespective of second-line tests. Non-invasive markers of fibrosis have not been sufficiently validated in children to recommend routine use in clinical practice.

Of note, recent guidance by NICE on NAFLD proposed single testing with serum ELF measurements without recourse to algorithms or other diagnostic modalities,^62^ on the basis of cost-effectiveness analyses. This recommendation was noted and carefully considered during the drafting of this guideline. On balance, however, it was felt that the evidence for diagnostic tests, and their use in combination in NAFLD, was still evolving and thus this guidance took a broader view setting out a pathway structure with a range of options. This view is endorsed by the EASL-ALEH guidelines for the non-invasive assessment of liver disease.^63^

***Recommendation 7: Adults with NAFLD should undergo risk stratification to determine their extent of liver fibrosis.***

- ***First-line testing should use either FIB-4 or NAFLD Fibrosis Score – see*** table 3  ***(level 2b, grade B). Calculation facilities for FIB-4 and NFS should be incorporated in all primary care computer systems. (level 5, grade D)***
- ***Second-line testing requires a quantitative assessment of fibrosis with tests such as serum ELF measurements or Fibroscan/ARFI elastography. (level 2b, grade B)***
- ***We recommend that hepatologists at a local level champion this idea and discuss it with commissioners of health to deal with the burden of liver disease in their area*** figures 1 and 2.

### ARLD

**Figure 3:**
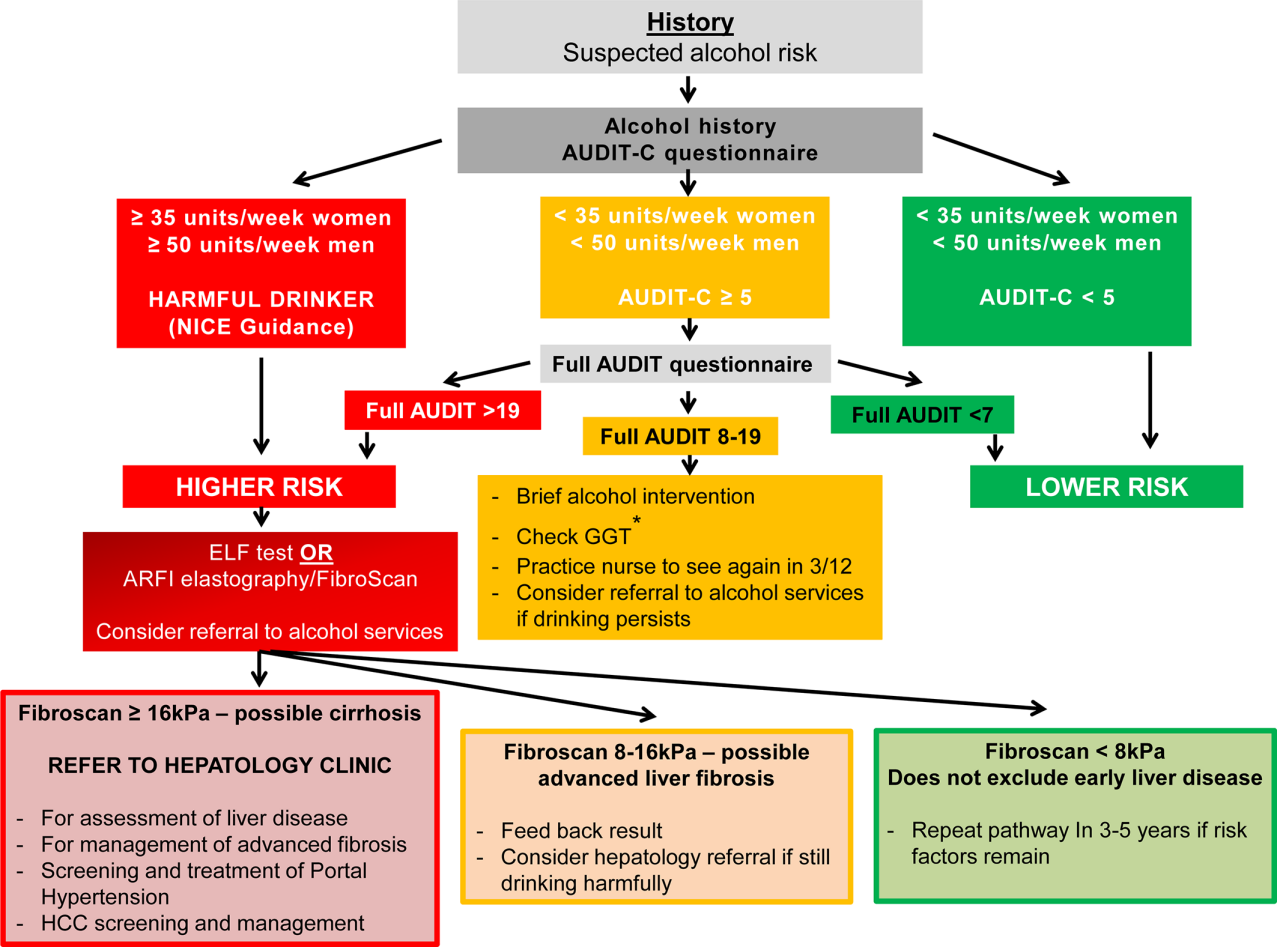
Alcohol-related liver disease algorithm. In patients in whom alcohol is suspected to be the main injurious factor, the extent of consumption influences early decision-making. For those drinking at harmful levels, ≥35 units/week women and ≥50 units/week men, an assessment of liver fibrosis is the critical next step. For other patients, administration of the AUDIT C questionnaire alongside brief intervention is recommended initially. For patients who continue to drink at hazardous levels consideration should be given to assessment as for the higher-risk category according to liver fibrosis evaluation. This is particularly important for those with a GGT of >100 U/L. Cut-off points for ARFI vary according to manufacturer and thus should be tailored to the device used. ARFI, acoustic radiation force impulse; ELF, enhanced liver fibrosis; GGT, γ-glutamyltransferase; HCC, hepatocellular carcinoma.

Alcohol-related cirrhosis is the the most common cause of liver-related mortality in Western populations. The majority of patients with this condition are heavy daily drinkers, with a median alcohol consumption of around 120 units/week.^2^ Notably, 25% of the population drink more than recommended guidelines (≤14 units/week), with 10% drinking twice as much and 1.4% drinking more than 75 units/week.^3^ The relationship between alcohol consumption and liver cirrhosis is exponential; at 20 units/week the relative risk is approximately 3, whereas at 80 units/week it is 30.^1^ There is also a synergy between alcohol intake and obesity; when body mass index (BMI) is >35, the risk of liver disease doubles for any given alcohol intake (see figures 1 and 3).

The aim of treatment in ARLD is for the patient to stop drinking harmfully, and this usually means complete abstinence.^8^ Referral to alcohol services should be undertaken for those patients with alcohol dependency as defined by an AUDIT score of >19, and kept in mind for those patients with an AUDIT score of ≥8. Importantly, a liver diagnosis in itself can be a highly effective intervention to change behaviour, and may be all that is required in many cases.^9^ However, current provision of such brief interventions in primary care is inconsistent and should be given greater priority.^64^

***Recommendation 8: Consider referral to alcohol services for all adults with ARLD with evidence of alcohol dependency as defined by an AUDIT score of >19. (level 3b, grade C)***

Normal liver blood tests do not rule out advanced liver fibrosis and cirrhosis, and so different approaches have been adopted/recommended for the identification of the at-risk patient. The Lancet Commission recommended the use of AUDIT-C as a first-line screening tool in high-risk groups followed by the full 10-item AUDIT to determine when to look for liver disease.^1^ The recent NICE guideline (NG50)^65^ on cirrhosis recommended a cut-off point of 50 units/week for men and 35 units/week for women, above which Fibroscan/ARFI elastography is recommended to detect cirrhosis and advanced fibrosis.^66 67^ Patients with cirrhosis require screening for oesophageal varices and hepatocellular carcinoma.

***Recommendation 9: Harmful drinkers should undergo risk stratification with clinical assessment and Fibroscan/ARFI elastography. Adults should be referred to secondary care if there is evidence of advanced liver disease (features of cirrhosis or portal hypertension on imaging or from blood tests) and/or Fibroscan reading is >16 kPa (if available). (level 2b, grade B)***

Patients flagged up with the AUDIT-C but drinking <35 units/week (women) and <50 units/week (men), respectively, should proceed to the full AUDIT questionnaire as detailed in figure 3. If GGT is elevated (>100 U/L) then consideration should be given to an assessment of liver fibrosis, as for the higher-risk group.

***Research Recommendation 2: Further evidence is required to establish the most cost-effective approach to identify patients with ARLD and NAFLD at risk of having advanced liver fibrosis.***

### Approach to a patient with abnormal liver blood tests and a negative extended liver aetiology screen

When the extended liver aetiology screen is negative (table 2), including an abdominal USS, it is important to re-examine the history to exclude potential drug-induced aetiologies, including over-the-counter preparations and any potential recreational/herbal drug use.^68 69^ In a UK primary care study of 1118 adults no cause was found in 45%, although many of these adults had metabolic risk factors and were likely to have NAFLD,^4^ and it is important to recognise that ultrasound is only sensitive for steatosis when hepatocytes are more than 30% steatotic so patients with milder steatosis might have a normal USS.^70^ Therefore patients with raised ALT and/or GGT levels who are obese and/or have metabolic risk factors may still have NAFLD despite a normal USS. Such patients should be assessed in accordance with the NAFLD fibrosis algorithm. To further risk stratify this group of patients offer a second- line test for fibrosis such as Fibroscan/ARFI elastography or ELF test as shown in figures 1 and 2. Those patients with no NAFLD risk factors (eg, type 2 diabetes mellitus (T2DM), BMI >25, dyslipidaemia and hypertension) and with persistently elevated liver enzymes should be considered for referral to a local specialist for further evaluation. For example, in autoimmune liver disease there may be no autoantibodies detected, and in some cases immunoglobulins may also be normal—as a result, some entirely treatable conditions may be overlooked. For children, assessment of fibrosis will be performed after referral to secondary care, and may include Fibroscan/ARFI elastography or biopsy depending on local practice and guidelines.

***Recommendation 10: Adults with abnormal liver blood tests, even with a negative extended liver aetiology screen and no risk factors for NAFLD, should be referred/discussed to a gastroenterologist with an interest in liver disease/hepatologist for further evaluation (*** figure 1 ***). (level 4, grade C)***

## Applicability

This guideline has provided advice on the pathways and tools to be used to best manage patients with abnormal liver blood tests. The pathways will be freely disseminated and incorporated into primary care IT systems to allow automatic calculation of risk scores when appropriate, to ensure recommendations can be put into practice.

Facilitators of the guideline will include specialist societies—in particular, the Royal College of General Practitioners, the British Liver Trust and the British Society of Gastroenterology. Barriers to use include access to the guideline and its potential complexity. This has been addressed by inclusion of all relevant stakeholders, a simplified set of pathway figures and a comprehensive dissemination plan.

The potential resource implications of applying the recommendations have been considered, and will be formally evaluated after adoption. At present, investigation and referral of patients with abnormal liver blood tests is variable, resulting in both unnecessary referral of patients and missing of significant liver disease. This pathway may rationalise the approach to abnormal liver blood tests and reduce healthcare expenses. An example of this is in Lambeth, where such a pathway has been successfully introduced, reducing referral of patients with NAFLD and minimal fibrosis.

## Monitoring and/or auditing criteria

1. Formal adoption of this referral pathway for abnormal liver blood tests within each Clinical Commissioning Group (CCG) and updated annually. Data to be expressed as number of CCGs having adopted the pathway as a percentage of all CCGs.
2. Review number of referrals for abnormal liver blood tests from each CCG per year and audit the number that had followed the pathway. Data to be expressed as a percentage of referrals.
3. Proportion of patients with NAFLD or ARLD who have an assessment of liver fibrosis, as evaluated by FIB-4, NFS, ELF, Fibroscan and/or ARFI, in their records. Data to be expressed as a percentage of patients coded with NAFLD or ARLD on GP list.
